# Immune and hemorheological changes in Chronic Fatigue Syndrome

**DOI:** 10.1186/1479-5876-8-1

**Published:** 2010-01-11

**Authors:** Ekua W Brenu, Donald R Staines, Oguz K Baskurt, Kevin J Ashton, Sandra B Ramos, Rhys M Christy, Sonya M Marshall-Gradisnik

**Affiliations:** 1 Faculty of Health Science and Medicine, Population Health and Neuroimmunology Unit, Bond University, Robina, Queensland, Australia; 2 Faculty of Health Science and Medicine, Bond University, Robina, Queensland, Australia; 3 Queensland Health, Gold Coast Population Health Unit, Southport, Gold Coast, Queensland, Australia; 4 Department of Physiology, Akdeniz University Faculty of Medicine, Antalya, Turkey

## Abstract

**Background:**

Chronic Fatigue Syndrome (CFS) is a multifactorial disorder that affects various physiological systems including immune and neurological systems. The immune system has been substantially examined in CFS with equivocal results, however, little is known about the role of neutrophils and natural killer (NK) phenotypes in the pathomechanism of this disorder. Additionally the role of erythrocyte rheological characteristics in CFS has not been fully expounded. The objective of this present study was to determine deficiencies in lymphocyte function and erythrocyte rheology in CFS patients.

**Methods:**

Flow cytometric measurements were performed for neutrophil function, lymphocyte numbers, NK phenotypes (CD56^dim^CD16^+ ^and CD56^bright^CD16^-^) and NK cytotoxic activity. Erythrocyte aggregation, deformability and fibrinogen levels were also assessed.

**Results:**

CFS patients (*n *= 10) had significant decreases in neutrophil respiratory burst, NK cytotoxic activity and CD56^bright^CD16^- ^NK phenotypes in comparison to healthy controls (*n *= 10). However, hemorheological characteristic, aggregation, deformability, fibrinogen, lymphocyte numbers and CD56^dim^CD16^+ ^NK cells were similar between the two groups.

**Conclusion:**

These results indicate immune dysfunction as potential contributors to the mechanism of CFS, as indicated by decreases in neutrophil respiratory burst, NK cell activity and NK phenotypes. Thus, immune cell function and phenotypes may be important diagnostic markers for CFS. The absence of rheological changes may indicate no abnormalities in erythrocytes of CFS patients.

## Background

Persistent unrelenting fatigue affects individuals across all ages worldwide and severe forms of prolonged fatigue may be diagnosed as Chronic Fatigue Syndrome (CFS) usually accompanied by other disabling symptoms. CFS is a heterogeneous multifactorial disease characterised by severe fatigue and an inability to function at optimal levels [1]. The multifactorial nature of this disease is due to the multiple causal factors associated with the disorder [2]. CFS by definition is a new onset of prolonged persistent fatigue enduring for over a period of 6 months or more, with the presence of at least four of the following symptoms; impaired short term memory or concentration, sore throat, tender cervical or auxiliary lymph nodes, multijoint pain with no indication of swelling or redness, severe headaches, unrefreshing sleep and postexertional malaise with a duration of 24 hours or more. Psychiatric disorders such as melancholic depression, substance abuse, bipolar disorder, psychosis and eating disorders are excluded when diagnosing patients based on this definition [3].

To date, the exact mechanism(s) of CFS remains elusive however immune deficiencies particularly in lymphocytes function and number have been observed as a potential factor. Importantly, consistent decreases in NK cytotoxic activity have been observed among different populations of CFS patients [4-7]. Some studies have suggested that these decreases in NK function may involve low levels of granzymes, perforin proteins and increases in the expression of the granzyme gene *GZMA *[6,8]. Although NK subsets, have been examined to some extent in CFS [4,9,10], these findings have not necessarily elucidated the role of CD56^bright^CD16^negative(neg) ^NK and CD56^dim^CD16^postive(pos) ^NK phenotypes in CFS. NK cells and their subsets are important in immune regulation and pathogen lysis. CD56^bright^CD16^neg ^NK cells preferentially secrete high levels of cytokines and have limited cytotoxic function while CD56^dim^CD16^pos ^NK cells are mainly cytotoxic [11]. Moreover, phagocytes such as neutrophils have received little attention, only one study has revealed that neutrophils in CFS are more prone to apoptosis, this was heightened by the existence of large quantities of TGFβ1[12].

The multifactorial and heterogeneous nature of CFS suggests changes in other blood indicators, such as erythrocytes. Some CFS patients demonstrate alterations in blood flow, erythrocyte rheology and erythrocyte morphology [13-17]. Abnormally shaped erythrocyte may present itself in the form of nondiscocytic, stomatocytic or cup formed erythrocyte [18]. Additionally, reductions in erythrocyte width and mass, and changes in platelet aggregation have also been detected in some CFS patients [13,16]. Plasma proteins such as fibrinogen which influence erythrocyte rheology are elevated in some CFS cases, and this may be related to impaired coagulation [19] however, an association between erythrocyte aggregation and fibrinogen levels in CFS is not presently known. Alterations in erythrocyte rheology may persist in CFS, these observations although indicative of indirect changes in deformation and aggregation suggests the need for further investigations to confirm the possible link between immune function and rheology in CFS.

Hence, the objective of this study was to examine immune function and rheological properties of peripheral blood cells. This study investigated NK abnormalities in CFS to confirm those of other studies. NK phenotypes, NK cytotoxic activity, neutrophil function, lymphocyte numbers, fibrinogen levels and erythrocyte rheology were measured in CFS patients. The CFS data were compared to aged and sex matched group of health volunteers.

## Materials and methods

### Participants

The present study was approved by Bond University Ethics Committee (RO852). Collection of venous blood was performed following consent from participants. Informed consent was prepared in accordance with the Bond University Research Consultancy Service and protocol. The CFS cohort comprised of 10 CFS patients from a community based sample in New South Wales and Queensland, Australia and 10 healthy aged and sex matched participants from a community local area. CFS patients were chosen after completion of a questionnaire adapted from the CDC 1994 CFS case definition [3], where the duration of CFS in our patient cohort was more than 5 years. Peripheral blood samples were analysed for total lymphocytes, NK activity, NK phenotypes, neutrophil function, erythrocyte deformability, erythrocyte aggregation and fibrinogen concentration.

### Lymphocytes assay

Peripheral blood lymphocyte subsets were assessed using fluorochrome-conjugated monoclonal antibodies from the Simultest IMK-Lymphocyte kit (BD Biosciences, San Jose, CA), specific for lymphocytes as previously described [20]. A fluorescence-activated cell sorting (FACS Calibur) flow cytometer (Becton Dickinson Immunocytometry Systems, San Jose, CA) was used to determine lymphocyte subsets, CD3+/CD19 (B cells), CD3+ (T cell), CD3+/Cd4+ (T-helper cells), CD3+/CD8+ (T-cytotoxic, T suppressor), CD3-/CD16+/CD56+ (Natural Killer cells).

### Assessment of NK lymphocyte activity

NK cytotoxicity was performed as previously described [21]. Briefly, NK cells were isolated from whole blood via density gradient centrifugation using ficoll-Hypaque (GE Healthcare). NK cells were labelled with 0.4% PKH-26 (Sigma, St Louis, MO). NK cells were resuspended at a final concentration of 5 × 10^6^cells/mL. The K562 cell line was used as the target cells at a concentration of 1 × 10^5^cells/mL. K562 cells were cultured with NK cells in RPMI-1640 culture media (Invitrogen, Carlsbad, CA) for 4 hours in 37°C incubator with 5% CO_2_, at an effector (NK) to target (K562) ratio of 25:1 with a control sample containing only K562 cells. Apoptosis was measured via flow cytometry, using Annexin V-FITC conjugated mAB and 7-AAD reagent (BD Pharmingen, San Diego, CA) according to the manufacturer's instructions. Percent lysis of K562 cells were calculated as previously described [21].

### Quantification of NK phenotypes

To assess the levels of NK phenotypes in CFS patients and healthy controls, NK lymphocytes were isolated from whole blood according to manufacturer's instructions using RosetteSep Human Natural Killer cell Enrichment Cocktail (StemCell Technologies, Vancouver, BC), containing micro-beads that negatively select for only NK cells and ficoll-hypaque density centrifugation. Samples were washed twice with PBS and labelled with mAB CD56-FITC (BD Bioscience, San Jose, CA) and CD16-PE (BD Bioscience, San Jose, CA) according to manufacturer's specifications and analysed on flow cytometer.

### Neutrophil function test

Immune response to pathogens was measured in granulocytes from lithium heparinised blood where phagocyte activity and respiratory burst was examined using the Phagotest and Phagoburst kit (Orpegen Pharma GmbH, Heidelberg, Germany) respectively as specified by the manufacturer. In summary, to determine phagocytosis, blood samples were mixed with FITC-labelled opsonised *E.coli *and incubated for 10 minutes in 37°C water bath or on ice at 0°C. Quenching solution was added to remove the FITC from the *E.coli*. Intracellular oxidation was performed by incubating heparinised whole blood in phorbol 12-myristate 13-acetate (PMA) for 10 minutes at 37°C. Dihydrohodamine (DHR) was then added to the samples followed by an incubation period of 10 minutes at 37°C. DHR was used as it is an indicator of neutrophil respiratory burst [22]. Samples were analysed on the flow cytometer.

### Measurement of erythrocyte aggregation and fibrinogen concentration

Erythrocyte aggregation was performed using the Myrenne aggregometer (Myrenne GmbH, Roetgen, Germany) in autologous plasma and 3% dextran solution (70 kDa; Sigma, St. Louis, MO) as previously described [23,24]. This method generates two distinct measures of erythrocyte aggregation at stasis (M_0_) and at a low shear (M_1_) after a shear rate of 600 s^-1^. Erythrocyte aggregation indices were determined at hematocrit of 40% at room temperature. Fibrinogen analysis was determined using blood mixed with sodium citrate solution. Samples were centrifuged at 1200 rcf for 10 minutes, platelet-poor plasma was collected and stored at -80°C for later analysis. Plasma fibrinogen was assessed by the CLAUSS method [25] using a STA-Compact analyser (Diagnostica Stago, Asnieres, France) where the intra-assay coefficient of variation was 2.64% and the inter-assay coefficient of variation was 2.82%.

### Erythrocyte deformability measurement

Deformability of erythrocyte was performed as previously described [26]. Blood samples were mixed with 0.99% RheoScan-D reagent (Incyto, Korea) and analysed on the RheoScan-D ektacytometer (Sewon Meditech, Korea). The elongation index was measured between shear stresses of 0.5 to 20 Pa. Shear stress for half-maximal deformation (SS_1/2_) and the maximum elongation index (EI_max_) was deduced using Lineweaver-Burk analysis. Measurements were carried out within 6 hours of blood collection and performed at room temperature (25°C).

### Statistical analysis

Statistical significance between the two subject groups was determined for all data using the independent sample *t *test. The data are represented as mean ± standard error of the mean (SEM).

## Results

### Distribution of leukocyte subsets

**Figure 1:**
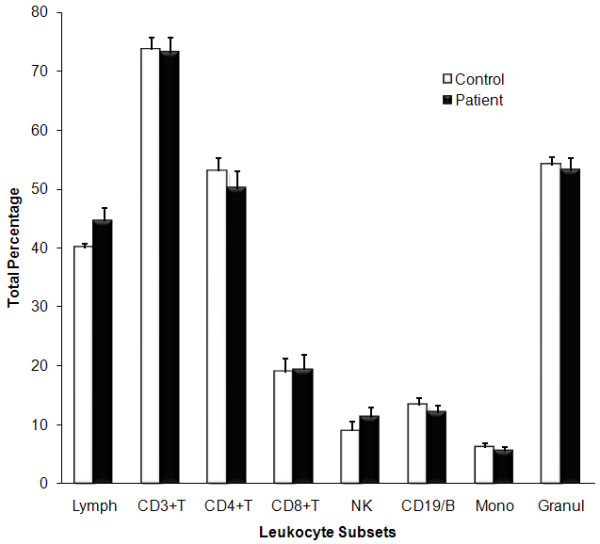
**Distribution of total leukocyte percentage in peripheral blood**. The percentage distribution of lymphocytes subsets in peripheral blood samples of CFS patients (Black bars; n = 10) and healthy controls (White bars; n = 10) was measured using the flow cytometer. Total lymphocytes, monocytes and granulocytes were performed using coulter analysis of full blood counts. All samples were analysed within six hours of collection. Leukocyte gate was used in determining the distribution of the various lymphocyte subsets. All values are presented as % means ± SEM.

The total number of circulating leukocytes in CFS patients and control participants were comparable. There was not distinct statistical difference in the percentages of B (CD3-/CD19+), T (CD3+/CD19-), CD4+T (CD3+/Cd4+), CD8+T (CD3+/CD8+) and NK (CD3-/CD56+/CD16+) lymphocytes (Figure 1). Additionally total circulating monocytes and granulocytes did not differ between groups.

### Altered distribution of NK phenotypes

**Figure 2:**
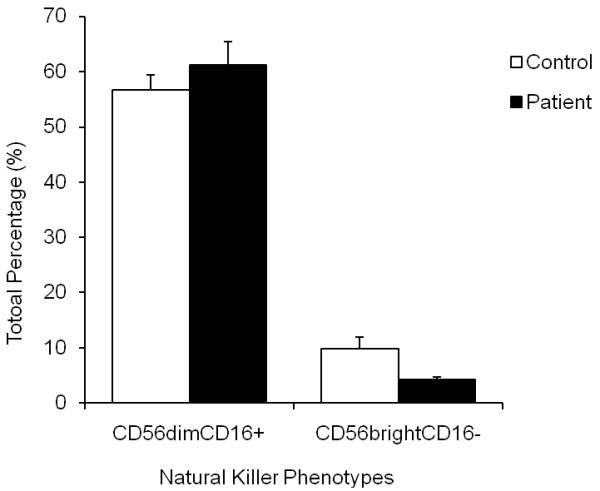
**Determination of NK cell phenotypes in whole blood samples**. NK cell phenotypes, CD56^dim^CD16^+ ^and CD56^bright^CD16^-^NK cells were determined by flow cytometry after separation from whole blood from CFS patients (white bars; n = 10) and control subjects (black bars; n = 10). The plots shown are gated on NK lymphocyte population. Data are the mean ± SEM. the symbol (*) denotes statistical significance.

The total number of NK phenotypes specifically CD56^bright^CD16^- ^and CD56^dim^CD16^+^NK cells were determined by flow cytometry. CD56^bright^CD16^- ^NK lymphocytes were significantly reduced (*P *< 0.05) in CFS patients (4% ± 0.5) compared to controls (10% ± 2.1) (Figure 2). CD56^dim^CD16^+ ^did not statistically differ between groups, as shown in Figure 2.

### Decreased NK cytotoxic activity

NK cytotoxic activity was measured by assessing the ability of NK lymphocytes from the healthy subjects and the control group to induce apoptosis in K562. The percentage lysis for the healthy subjects and the CFS patients were significantly different. After 4 hours of incubation, NK cytotoxic activity was significantly lower in CFS patients compared to the healthy controls (13.6% ± 5.1 and 34.3% ± 6.6 SD, respectively, *P *< 0.05). There were more viable cells (Annexin V-FITC negative/7-AAD negative) in the patient sample compared to the healthy control group.

### Impaired neutrophil function

**Figure 3:**
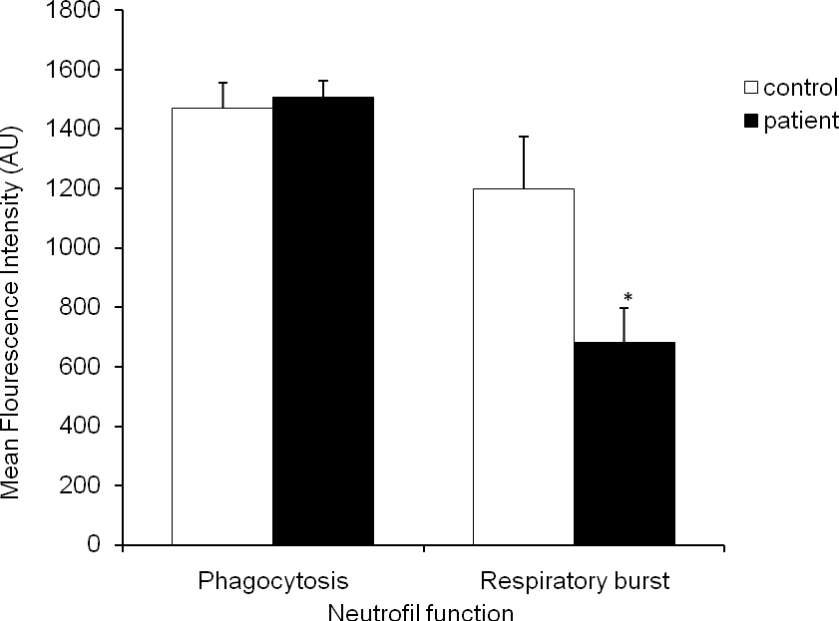
**Examination of neutrophils function in the presence of *E. coli***. The action of neutrophils phagocytic activity and respiratory burst function were compared between the two subject groups; CFS patients (black; n = 8) and controls (white; n = 8). RBF is respiratory burst while PF is phagocytic activity. Results represent the mean ± SEM the symbol (*) denotes statistical significance.

Phagocytosis in neutrophils was measured via flow cytometer using the Phagotest kits, where neutrophils after phagocytising FITC-labelled *E.coli *are FITC-positive. In neutrophils of healthy subjects and CFS patients, phagocytosis of *E. coli *was not significantly different between CFS patients (1507 arbitrary units (AU) ± 54) and healthy subjects (1471 AU ± 85) (Figure 3). Intracellular oxidation, that is, the ability of the neutrophils to produce reactive oxygen species after intake of *E. coli *was determined using the Phagoburst kit. As illustrated in figure 3, in the healthy subjects (1199 ± 177 mean fluorescence intensity (MFI)), a significantly higher amount of neutrophils are affirmative for intracellular oxidation of *E. coli*, while in the CFS patients (681 ± 115 MFI) significantly lower levels of neutrophils were positive for oxidative burst after phagocytising the *E. coli *(*P *< 0.05).

### Erythrocyte aggregation and deformability

**Figure 4:**
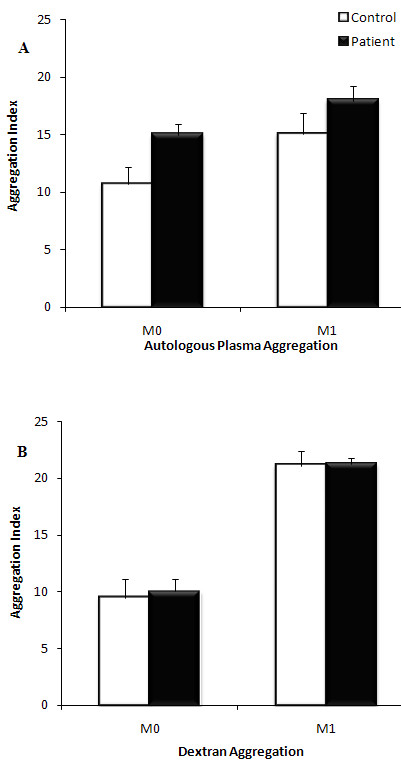
**Assessment of erythrocyte aggregation in autologous plasma (A) and dextran solution (B)**. Peripheral blood samples from CFS patients (black; n = 10) and healthy controls (white; n = 10) assessed on measures of aggregation at stasis (M_0_) and at low shear rate (M_1_). Samples were measured after adjustment of hematocrit to 40% (A) following which they were washed and suspended in 3% dextran solution with a hematocrit 40% hematocrit adjustmnent (B). Samples were analysed within 12 hours of blood collection. Results are represented as mean ± SEM.

**Figure 5:**
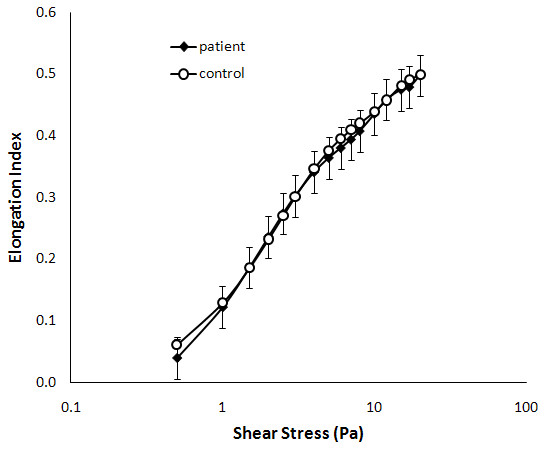
**Assessment of erythrocyte deformability in CFS**. Peripheral blood samples from CFS patients (black; n = 6) and healthy controls (white; n = 6) were assessed. Deformability was assessed at shear stresses from 0.5-20 Pa. The mean ± SEM are represented on the graph

**Figure 6:**
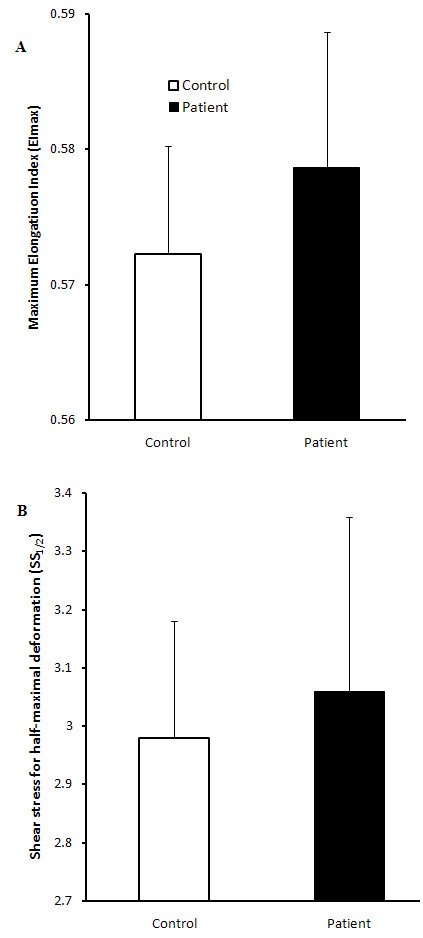
**Erythrocyte deformability after determination of EI_max _and SS_1/2_**. EI_max _(A) and SS_1/2 _(B) of CFS patients (black; n = 6) and healthy controls (white; n = 6) were not significantly different. The values are the mean ± SEM of the two groups.

Erythrocyte aggregation at the end of suspension in autologous plasma was not significantly different (Figure 4) between groups at both M_0 _(stasis) and M_1 _(low shear). Erythrocyte aggregation for cells washed and resuspended in 3% dextran solution was also not significantly different between groups, either at stasis or at low shear stress (Figure 4). Although plasma fibrinogen levels was markedly higher in CFS patient (3.59 ± 0.38 SD) compared to healthy subjects (2.95 ± 1.11 SD) this did not attain statistical significance. Similarly, there was no significant change in deformability between groups. Deformability was measured based on the *EI *of the whole erythrocyte from a shear stress of 0.5-20 Pa. The average *EI *at shear stresses from 0.5-20 Pa are represented in Figure 5. No significant differences were noted at any of these shear stresses for six individuals from each group. Similarly, SS_1/2 _and EI_max _did not change significantly between the two groups (Figure 6).

## Discussion

The primary objective of this study was to determine immunological and rheological characteristics of fatigue related conditions such as Chronic Fatigue Syndrome (CFS). This is the first study to confirm significant changes in NK phenotypes in CFS particularly decreases in CD56^bright^CD16^- ^NK cells from preferentially isolation of NK cells from whole blood. Similar to other findings NK cytotoxic activity was also decreased. This study has illustrated for the first time significant reductions in neutrophil respiratory burst in CFS patients. However, it is apparent from these findings that CFS patients have normal lymphocyte numbers and normal erythrocyte rheology, particularly aggregation and deformability, perhaps indicating that the symptomatology of CFS does not entail aberration in erythrocyte activity. CFS may potentially involve immune dysfunction where these defects may entail lymphocyte activities and other related immune molecules.

NK phenotypes have been shown to be differentially expressed with no consistency in the subtype that may be altered in expression in CFS [4,9,10]. In our data significant decreases in CD56^bright^CD16^- ^NK cells were noted among CFS participants this may be related to impaired chemotaxis. CD56^bright^CD16^- ^NK preferentially expresses the chemokine receptor 7 (CCR7) and higher levels of chemokine receptor (CXCR) 3 in response to chemokines CCL19, CCL21 and CXCL10, CXCL11 respectively [27,28]. These chemokines are released from pathogens and secondary lymphoid organs allowing the migration of CD56^bright^CD16^- ^NK to the epithelia, periphery and other lymphoid organs during an inflammatory response [28,29]. Thus, impaired chemokine receptors may possibly affect the migration of these subsets of NK cells. Data from gene expression studies in CFS have indicated differential expression in the chemokine receptor *CXCR4 *[30], whose protein CXCR4 is expressed on both subtypes of resting NK cells [31,32]. Since no significant changes were observed in the number of CD56^dim^CD16^+ ^NK cells between groups, it is likely that poor chemokine receptor function affected the CD56^bright^CD16^- ^NK migration to the periphery. Interestingly, activated CD56^bright^CD16^- ^NK cells also produce chemokines CXCL8, CCL4, CCL5 and CCL22 [33,34]. CXCL8 is required for the migration and recruitment of CD56^dim^NK cells [35] changes in their expression can affect the recruitment of CD56^bright^CD16^- ^NK cells and limit immune response to either foreign or native pathogens with possible impairments in other immune cell activation [36].

NK cells are responsible for producing cytokines such as interferon (IFN)-γ (NK cells are the main producers), tumour necrosis factor (TNF)-α, granulocyte macrophage colony-stimulating factor (GM-CSF), interleukin (IL)-10, IL-8 and IL-13 required for the activation and maturation of macrophages, dendritic cells and T cells and immunosuppression [37]. IFN-γ release activates the Fas ligand cytotoxic mediated pathway on NK cells which produces a cascade of caspase signalling domains that effectively lyse the target cell [38]. TNF-α once produced by CD56^bright^CD16^- ^NK can either bind directly to TNF-α receptors on the infected cell and induce apoptosis of the target cell or initiate TNF-related apoptosis-inducing ligand (TRAIL) on NK cells thus activating caspase and inducing cytotoxic activity [39]. CD56^bright^CD16^- ^NK cells are therefore important for NK cytotoxic activity and a correlation exists between these subtypes of NK cells and NK cytotoxic activity.

Reduced NK CD56^bright^CD16^- ^NK cells have also been observed in patients with coronary heart disease, allergic rhinitis and juvenile rheumatoid arthritis, in all cases NK cytotoxic activity was also reduced [40,41]. The reduction in cytotoxic activity was explained by a reduction in IFN-γ producing CD56^bright^CD16^- ^NK cells which led to poor cytotoxic activation. Additionally changes in IFN-γ production are associated with recurrent infections, production of adequate levels of IFN-γ during initial infection are crucial for protection against subsequent infections [42]. Importantly, CD56^bright^CD16^- ^NK cells are critical for early innate and adaptive immune response as they are more proliferative and exert immunoregulatory effects on other lymphocytes through the cytokines and chemokines they release [43].

Neutrophils are essential cells in the innate immune system. They primarily function to engulf and lyse pathogens via phagocytosis and respiratory burst [44]. Effective lysis occurs during respiratory burst where the oxidation of super peroxides by NADPH results in the production of a cascade of reactive oxygen species, which cumulatively eliminate the pathogen. Decreases in neutrophil function are indicative of impaired immune function in CFS. Only one study to date has demonstrated that neutrophils in CFS patients are highly apoptotic with an increase in TGF-β and TNFR1 [12]. Delayed or limited apoptosis correlates with an increase in respiratory burst [45], thus a situation where decreases in respiratory burst persist may likely be an indicator of elevations in apoptotic neutrophils. This potentially increases the life of bacteria and other pathogens in the body as they are not efficiently lysed owing to limited intracellular oxidative processes. Diminishing levels of CD56^bright^CD16^- ^NK cells may limit the production of TNFs, cytokines required for activation of respiratory burst in neutrophils. TNF-α and GM-CSF, produced by CD56^bright^CD16^- ^NK, are important for the induction of superperoxide thus priming the neutrophils for respiratory burst [46].

Decreases in NK cytotoxic activity have been consistently reported in previous studies [4,6]. Decrease in NK activity may be correlated with decreases in perforin and granzyme production [6] and changes in granzyme gene (*GZMA*) expression [8]. These deficiencies in NK activity may increase viral load in CFS, incidentally a recent study observed increases in xenotropic murine leukemia virus-related virus (XMRV) in peripheral blood samples of CFS patients [47]. These viruses may potentially alter aspects of the immune response such as cytotoxic activity thus promoting their survival in particular immune cells. NK cells and neutrophil deficiencies in CFS may be related to the presence of autoantibodies. Some of these autoantibodies are specific for proteins that may interact with immune cells have been detected in serum samples in CFS patients [48-50], however, these autoantibodies are yet to be detected against specific receptors expressed on immune cells or cellular lytic pathways.

There was no change in erythrocyte deformability or aggregation between groups, although other studies have confirmed changes in erythrocyte shape in CFS patients, particularly an increase in stomatocytes or lepotocytes [15,51]. Equally, the Lineweaver-Burk analysis did not indicate statistical significance between the two groups. The most likely consequence of these observations is the heterogeneity of CFS. Nonetheless, observable rheological changes are perhaps associated with the acute phase of CFS while these maybe absent during the chronic stages of the disorder [52]. Notably all CFS participants in this study were in the chronic phase. Thus, erythrocyte deformability and aggregation may not be distinct markers for CFS.

Given the paucity in CD56^bright^CD16^- ^NK cells among CFS patients in this study and their role in immunoregulation and activation, reduced CD56^bright^CD16^- ^NK cell numbers may be important in the pathomechanism of CFS, a disorder shown to be characterised by decreases in NK cytotoxic activity. Although changes in NK cell makers have been previously reported, a mechanism underlying diminishing NK cell markers and phenotypes has not yet been established. This mechanism may also involve changes at the genomic level which results in deficient cytokine and chemokine receptor expression. For example, alterations in RNA expression levels for CD56^bright^CD16^- ^NK receptors has been demonstrated in patients with Autism Spectrum Disorder where cytotoxic activity and NK cell numbers were also decreased when NK cells were stimulated by a pathogen [53]. Exposure to pathogens in the presence of differential expression of certain NK cytokine and chemokine receptor genes may trigger a decline in CD56^bright^CD16^- ^NK cells and NK cytotoxicity in CFS.

However, the heterogeneity and multifactorial nature of CFS suggests variations in molecular changes and cellular mechanisms among patients. Certain cytokines increase cytotoxic ability (IL-2) and IFN-γ production (IL-12 and IL-18) of CD56^bright^CD16^- ^NK [36], therefore a possible mechanism limiting the production of these cytokines and may adversely alter the role of CD56^bright^CD16^- ^NK during pathogen invasion and lysis. High levels of TFG-β also cause an increase in neutrophil apoptosis and this occurs in some cases of CFS [11]. Finally viral-specific infections may be necessary for NK deficiencies in CFS given that the Human Immunodeficiency Virus type 1 Viral Protein R (HIV-1 Vpr) upregulates TGF-β and decreases macrophage production of IL-12 causing a decline in cytotoxic activity and IFN-γ [54]. These mechanisms may be present in CFS and involve deficiencies in the ability of other leukocytes specifically macrophages and dendritic cells, to activate the NK cells [43].

## Conclusions

The information presented in this study confirms significant declines in immune function in CFS specifically in CD56^bright^CD16^- ^NK cell numbers, NK cytotoxicity and neutrophil respiratory burst. This is the first study to simultaneously assess innate immune function, phagocytosis and cytotoxic activity in CFS. The defects in innate immune function observed in this study potentially suggests an altered adaptive immune response in CFS and these may be important in understanding the pathomechanism of CFS. Further studies are however required to determine cytokine and chemokine expression in CFS patients. Neutrophil apoptosis in relation to respiratory burst, cytotoxic activity in CD8 T cells, perforin and granzyme production and CD4+T cell cytokine secretion in CFS patients are potential topics for future investigations. These studies will allow a comprehensive analysis of the overall immune function in CFS patients.

## Conflict of interest statement

The authors declare that they have no competing interests.

## Authors' contributions to the paper

EWB assessed and recruited patients and controls for study, performed NK cytotoxic activity, NK phenotype analysis and erythrocyte experimental assessments, all statistical analysis and wrote the manuscript. SBR performed the IMK lymphocyte and full blood count test. RMC performed neutrophil function analysis. DRS provided the patient cohort and reviewed the manuscript. KJA second principal investigator advised on methodology and reviewed the paper. OKB provided the methodology for erythrocyte aggregation and deformability. SMM-G primary principal investigator advised on methodology and reviewed the manuscript. Authors read and approved the manuscript.
